# Nomogram for Predicting In-hospital Mortality in Infective Endocarditis Based on Early Clinical Features and Treatment Options

**DOI:** 10.3389/fcvm.2022.882869

**Published:** 2022-04-27

**Authors:** Zhao-Jun Yu, Zhi Dou, Jing Li, Zhi-Jie Ni, Guo-Xing Weng

**Affiliations:** Department of Cardiovascular Surgery, Shengli Clinical Medical College of Fujian Medical University, Fujian Medical University, Fuzhou, China

**Keywords:** infective endocarditis, prognosis, nomogram, prediction model, surgery

## Abstract

**Aim:**

The aim of this study was to develop a nomogram based on early clinical features and treatment options for predicting in-hospital mortality in infective endocarditis (IE).

**Methods:**

We retrospectively analyzed the data of 294 patients diagnosed with IE in our hospital from June 01, 2012 to November 24, 2021, determined independent risk factors for in-hospital mortality by univariate and multivariate logistic regression analysis, and established a Nomogram prediction model based on these factors. Finally, the prediction performance of nomogram is evaluated by C-index, bootstrapped-concordance index, and calibration plots.

**Results:**

Age, abnormal leukocyte count, left-sided IE, right-sided IE, and no surgical treatment were independent risk factors for in-hospital mortality in patients with IE, and we used these independent risk factors to construct a nomogram prediction model to predict in-hospital mortality in IE. The C-index of the model was 0.878 (95% CI: 0.824–0.931), and the internal validation of the model by bootstrap validation method showed a prediction accuracy of 0.852 and a bootstrapped-concordance index of 0.53.

**Conclusion:**

Our nomogram can accurately predict in-hospital mortality in IE patients and can be used for early identification of high-risk IE patients.

## Introduction

Infective endocarditis (IE) refers to the inflammation of the heart valve or the inner lining of the ventricular wall caused by the invasion of bacteria, fungi or other microorganisms. It is a common inflammatory disease worldwide, with a global incidence of 1.8/100,000–9.7/100,000 (1). As the aging trend of the global population increases, the number of patients with degenerative valvular disease is increasing, and IE is the most serious and devastating complication of heart valve disease, with a high clinical mortality rate. The 2016 American Association for Thoracic Surgery (AATS) consensus reported that even with appropriate antimicrobial therapy and surgical interventions in current medical conditions, the in-hospital mortality rate of IE is still as high as 15–20% (2). The prognosis of IE is influenced by four main factors: the clinical characteristics of the patient, the presence of cardiac and non-cardiac complications, the type of microorganisms infected and the echocardiographic findings (3). Applying early clinical characteristics and laboratory testing to identify high-risk patients with IE for targeted treatment is an effective strategy to reduce mortality during hospitalization in patients with IE and is a significant problem for doctors.

In recent years, statistical prediction models have been widely used in the prognostic studies of clinical diseases. One of the prognostic prediction models is the nomogram. Its working method is to build a statistical prediction model to generate numerical probabilities of clinical events, then use the nomogram to display the outcomes of the statistical prediction model in simple graphics, and finally to make clinical decisions based on them. Nomogram is frequently utilized in the prognostic research of tumor patients due to its simplicity and convenience of usage. The effectiveness of the nomogram to predict prognosis in many tumors is much better than that of traditional TNM staging (4). The Nomogram prediction model has been shown in a number of studies to accurately quantify and evaluate the probability of clinical events in patients, with the predicted results matching the real world (5–7).

We hope that by constructing a Nomogram prediction model based on patients' early clinical data, we will be able to use the probabilities generated by the model predictions to risk-stratify patients with IE in the clinic, allowing clinicians to identify patients with a poor prognosis earlier and take more aggressive and targeted treatment measures to reduce mortality during hospitalization in patients with IE.

## Materials and Methods

### Study Population

We used the hospital electronic medical record system to get clinical data from patients who visited our hospital and were diagnosed or suspected of having infective endocarditis between June 1, 2012, and November 24, 2021. A total of 694 cases were retrieved. The modified Duke diagnostic criteria were used to screen the patients, and 383 individuals with IE who satisfied the duke diagnostic criteria were included (8). Inclusion criteria for this study were patients with a definite end point event during hospitalization, that is, those who were cured with antibiotics or surgical treatment during hospitalization [Criteria for IE cure: The criteria for drug cure is that after 4–6 weeks of antibiotic application, the patient's conscious symptoms are relieved or disappeared, no fever is manifested, the indexes of infection such as blood routine, C-reactive protein (CRP), Procalcitonin (PCT) are significantly decreased or returned to normal, and the patient's two consecutive blood culture tests are indicated to be negative. The echocardiogram showed that the cardiac vegetation was reduced or even disappeared. The criteria for surgical cure are that the structural abnormality of the heart is corrected, the heart valve function is good, and there is no cardiac vegetation, and other criteria are the same as those for drug cure], and those who did not improve with treatment and died during hospitalization. Exclusion criteria were patients who did not have sufficient evidence of an end point event during hospitalization, that is, those who refused treatment or were discharged without definite cure or death. Finally, 294 patients were included in the study, and clinical data from the start of the disease and therapeutic measures taken during hospitalization were obtained from the participants.

### Clinical Data Acquisition

The general information of the enrolled subjects included age, gender, height, weight, first symptoms, clinical symptoms, history of previous cardiac disease, history of hemodialysis, history of central venous cannulation, recent history of oral disease therapy and so on. Laboratory tests included blood routine, blood biochemistry, urine routine, CRP, PCT, peripheral blood cultures, cardiac valve tissue culture, chest X-ray, electrocardiogram (ECG), transthoracic echocardiography (TTE) and transesophageal echocardiogram (TEE). In order to reflect the early clinical characteristics of the patients, the hematological examination of patients who visited our hospital directly and were not treated in other hospitals was based on the first blood test after admission, and the results of the tests before the patient received antibiotics were used for those patients who visited other hospitals and were later referred to our hospital. The echocardiographic examination results shall be based on the first examination after admission. If a patient has both TTE and TEE examinations, the TEE will take precedence. Cardiac vegetation is defined by cardiac echocardiography as thrombotic masses with chaotic echoes and unstable movements independent of valves (9). Left-sided IE was defined if the vegetation were found in the mitral and aortic valves, or in the inner wall of the left atrium and left ventricle by echocardiography. If the vegetation is found in the tricuspid or pulmonary valve, or in the inner wall of the right atrium or right ventricle, it is defined as right-sided IE. Bilateral IE is defined if there is vegetation on both sides. Anti-infective medication therapy and surgical treatment are major factors impacting the in-hospital mortality of IE patients in clinical treatment. As a result, in addition to collecting early clinical characteristics of IE patients, we also collected the duration of antibiotic use and whether surgical treatment was administered during the patients' hospitalization, and these data will be used as key variables in the development of nomogram statistical prediction models.

### Construction and Evaluation of Nomogram Prediction Model

The factors that may influence mortality during hospitalization in patients with IE were first screened using univariate logistic regression analysis, and then the statistically significant components were added to a multivariate logistic regression analysis model. Finally, the screened independent risk factors were used to create a nomogram. A concordance index and calibration plots with bootstrap samples were used to assess the nomogram's performance. A concordance index is a numerical assessment of discriminative capacity. Calibration plots are graphic evaluations of predictive ability that compare observed probabilities with nomogram-predicted probabilities (10).

### Statistical Analyses

Statistical analysis was performed using Social Package for the Social Sciences (SPSS) version 26 software. Kolmogorov–Smirnov and Shapiro–Wilk tests were used to determine whether the distribution of variables was normal. Numerical data are expressed as means ± standard deviations (*x* ± *s*) or medians (interquartile range) depending on whether they conform to a normal distribution. Student's *t*-test or the Mann–Whitney U-test was used to evaluate the numerical data between groups. Categorical values are expressed as percentages. For compare groups of categorical data, the chi-square test or Fisher's exact strategy was applied. A *P*-value < 0.1 was considered statistically significant in univariate regression analysis. In multivariate regression analysis and data comparison between two groups, a *P*-value < 0.05 was deemed as statistically significant.

## Results

### Clinical Characteristics of the Study Population

**Figure 1:**
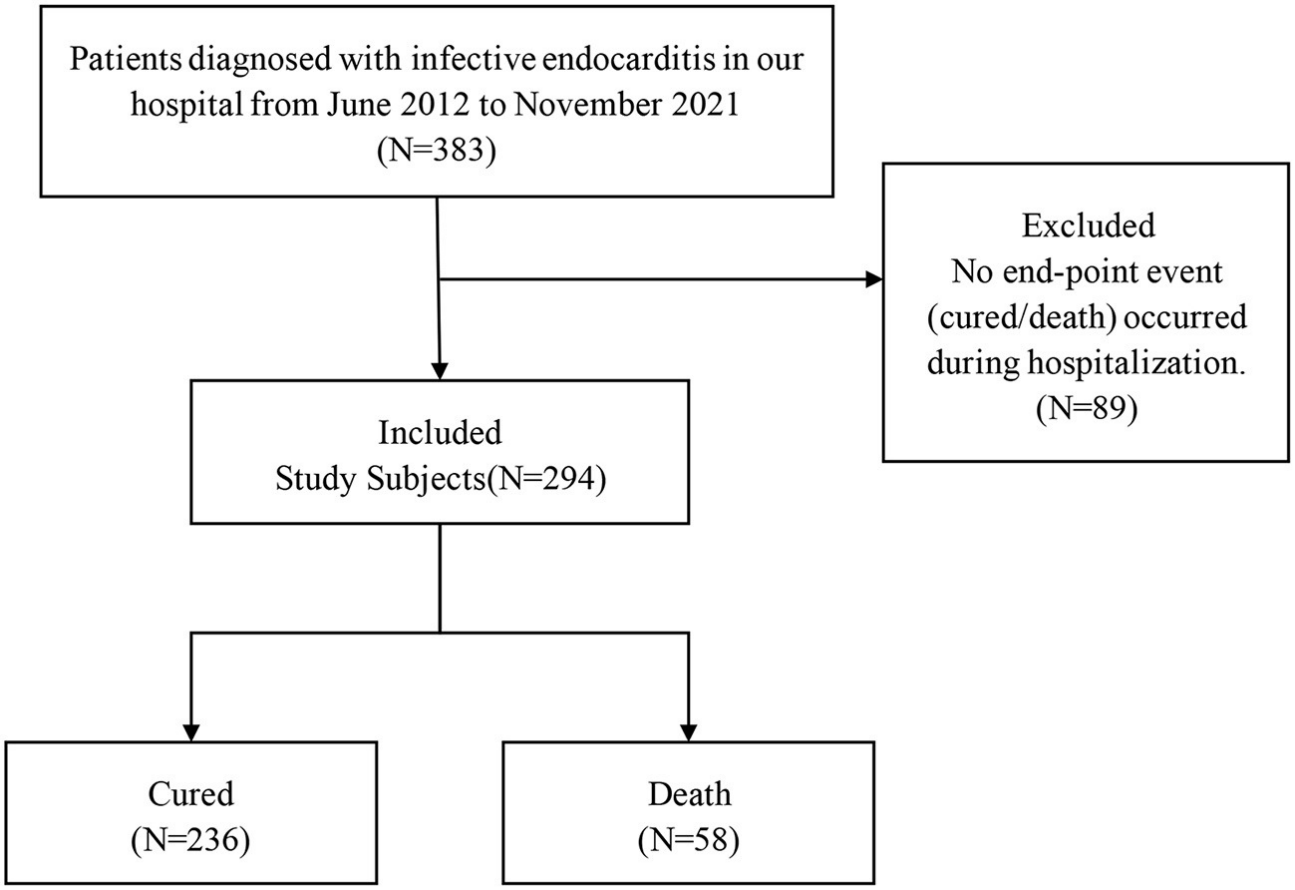
Study enrollment flowchart.

According to the inclusion and exclusion criteria of the study, a total of 294 study subjects were included, including 236 in the cured group and 58 in the death group (Figure 1).

To begin, we ran a general statistical analysis of the 294 study participants (Table 1). Among all the study subjects, the age group of 50–75 years old had the highest number of people with 139 people, accounting for 47.3%. Male patients made up two-thirds of the total, far more than female patients. The majority of the patients, 43.9%, had subacute onset, while 73 had acute onset, accounting for 24.8%. Fever is the most common symptom in most patients, with 64.3% of patients experiencing fever initially, followed by heart failure symptoms. After admission with blood cultures and cardiac ultrasonography, 17 patients with symptoms of cerebral infarction or vascular embolism were identified with infective endocarditis. A total of 171 individuals reported positive blood cultures, resulting in a 58.2% positive rate. One hundred and fifty one patients (88.3%) had Gram-positive bacterial infections, 14 patients (8.2%) had Gram-negative bacterial infections, and 6 patients (3.5%) had fungal infections among those with positive blood cultures. Fifty eight patients (19.7%) died while in the hospital, while 236 patients (80.3%) were cured and discharged after surgery or active anti-infective treatment. The individuals were separated into two groups based on the study outcomes: cured and death.

**Table 1:** Baseline data of the study population.

**Factors**	**Category**	**Number of cases**	**Percentage**
Age	≤ 25	21	7.1
	25–50	125	42.5
	50–75	139	47.3
	>75	9	3.1
Gender	Male	197	67.0
	Female	97	33.0
Disease-process	Acute	73	24.8
	Sub-acute	129	43.9
	Chronic	92	31.3
Primary symptoms	Fever	189	64.3
	Embolism symptoms	17	5.8
	Heart failure	74	25.2
	Others	14	4.8
Blood culture	Positive	171	58.2
	Negative	123	41.8
Pathogens	Gram-positive Bacteria	151	88.3
	Gram-negative Bacteria	14	8.2
	Fungus	6	3.5
Outcomes	Cured	236	80.3
	Death	58	19.7

We compared the cured and death groups to see if there were any risk variables that affected the study's outcomes (Table 2). Patients in the cured group were (47.75 ± 15.68) years old, whereas those who died were (58.84 ± 15.81) years old. There was a statistically significant difference in the overall mean age of the patients in the two groups (difference 11.10, 95 %CI 6.57–12.63, *t* = 4.82, *P* < 0.001). The proportion of patients with prosthetic valve or cardiac device in the cured group was 13.6%, and that in the death group was 27.6%. There was a statistical difference in the proportion of patients with prosthetic valve or cardiac device in the two groups (*P* = 0.010). The cured group had a median leukocyte count of 9.13 (4.36) × 10^9/^L, while the death group had a median leukocyte count of 11.83 (7.38) × 10^9^/L, with a statistically significant difference in the distribution of leukocyte counts between the two groups (z = 3.87, *P* < 0.001). Left-sided endocarditis accounted for 62.7% in the cured group and 77.6% in the death group, according to the results of a cardiac ultrasound examination, and there was a statistical difference in the proportion of left-sided endocarditis between the two groups (*P* = 0.033). During hospitalization, 85.2% of patients in the cured group and 72.4% of patients in the death group were on antimicrobial drugs for more than 6 weeks, with a statistical difference in the proportion of time on antimicrobial drugs between the two groups (*P* = 0.022). The proportion of patients in the cure group who received surgery was 86.4%, and that in the death group was 24.4%. There was a statistically significant difference in the proportion of patients in the two groups who received surgery (*P* = 0.022).

**Table 2:** Comparison of clinical characteristics and laboratory tests of the two groups.

**Items**	**All patients (*N =* 294)**	**Cured (*N =* 236)**	**Death (*N =* 58)**	***P*** **-value**
Age (Mean ± SD)	49.94 ± 16.29	47.75 ± 15.68	58.84 ± 15.81	<0.001
BMI (M, IQR)	21.80 (3.57)	21.48 (4.13)	22.2 (2.7)	0.384
Gender (*n*, %)
Male	197 (67.0)	159 (67.4)	38 (65.5)	0.788
Female	97 (33.0)	77 (32.6)	20 (34.5)	
Valvular disease (*n*, %)
Yes	79 (26.9)	58 (24.6))	21 (36.2)	0.073
No	215 (73.1)	178 (75.4)	37 (63.8)	
Congenital heart disease (*n*, %)
Yes	38 (12.9)	32 (13.6)	6 (10.3)	0.513
No	256 (87.1)	204 (86.4)	52 (89.7)	
Prosthetic valve or intracardiac device (*n*, %)
Yes	48 (16.3)	32 (13.6)	16 (27.6)	0.010
No	246 (83.7)	204 (86.4)	42 (72.4)	
Central venous catheters inserted (*n*, %)
Yes	13 (4.4)	8 (3.4)	5 (8.6)	0.168
No	281 (95.6)	228 (96.6)	53 (91.4)	
**Laboratory tests (M, IQR)**
Leukocyte count (×10^9^/l)	9.60 (4.83)	9.13 (4.36)	11.83 (7.38)	<0.001
Platelet count (×10^9^/l)	205.00 (128.00)	214.50 (128.00)	181.78 (133.00)	0.017
CRP (mg/l)	51.85 (68.16)	48.10 (63.56)	77.34 (70.49)	0.033
PCT (ng/ml)	0.37 (4.71)	0.33 (4.14)	0.80 (5.23)	0.173
Anemia (g/l) (*n*, %)
≥90	222 (75.5)	182 (77.1)	40 (69.0)	0.196
<90	72 (24.5)	54 (22.9)	18 (31.0)	
Blood cultures
Positive	171 (58.2)	137 (58.1)	34 (58.6)	0.937
Negative	123 (41.8)	99 (41.9)	24 (41.4)	
**Echocardiography**
Left-sided endocarditis
Yes	193 (65.6)	148 (62.7)	45 (77.6)	0.033
No	101 (34.4)	88 (37.3)	13 (22.4)	
Right-sided endocarditis
Yes	19 (6.5)	12 (5.1)	7 (12.1)	0.101
No	275 (93.5)	224 (94.9)	51 (87.9)	
**Treatment measures**
Duration of antibiotic use
≥6w	243 (82.7)	201 (85.2)	42 (72.4)	0.022
<6w	51 (17.3)	35 (14.8)	16 (27.6)	
Surgery
Yes	218 (74.1)	204 (86.4)	14 (24.4)	<0.001
No	76 (25.9)	32 (13.6)	44 (75.9)	

*BMI, body mass index; CRP, C-reactive protein; PCT, procalcitonin*.

### Logistic Regression Analysis of Factors Influencing In-hospital Mortality in IE

We performed logistic regression by incorporating characteristics that differed significantly between the cured and death groups, as well as variables in the clinic which might influence mortality from infective endocarditis during hospitalization (Table 3). Univariate logistic regression analysis found that age, embolic symptoms, previous heart valve disease, prosthetic valves or intracardiac devices, history of central venous cannulation, leukocyte count, PCT, left-sided IE, right-sided IE, duration of antibiotic use, and without surgery were associated with higher in-hospital mortality (*P* < 0.1). The above variables were included in a multivariate logistic regression model, and the results indicated that age (OR = 2.28, 95%CI 1.01–5.15, *P* = 0.048), Abnormal leukocyte count (OR = 2.46, 95%CI 1.12–5.36, *P* = 0.024), left-sided infective endocarditis (OR = 2.92, 95% CI 1.20-7.13, *P* = 0.019), right-sided infective endocarditis (OR = 6.58, 95% CI 1.63–26.53, *P* = 0.008), and without surgery (OR = 18.94, 95%CI 8.18–43.86, *P* < 0.001) were independent risk factors for mortality during hospitalization in patients with infective endocarditis (*P* < 0.05).

**Table 3:** Univariate and multivariate logistic regression analysis of in-hospital mortality in infective endocarditis.

**Factor**	**Classification and Description**	**Univariate analysis**			**Multivariate regression**		
		**OR**	**95%CI**	***P*** **-value**	**OR**	**95%CI**	***P*** **-value**
Age	(<50/≥50)	2.91	1.55–5.46	0.001	2.28	1.01–5.15	0.048
Gender	(Female/male)	1.09	0.59–1.99	0.788			
Clinical symptoms	Fever	0.87	0.47–1.60	0.646			
	Embolic symptoms	2.26	1.15–4.47	0.019			
	Heart failure	1.71	0.90–3.25	0.105			
**History of heart disease**
valve disease	(No/yes)	1.74	0.95–3.21	0.076			
congenital heart disease	(No/yes)	0.74	0.29–1.85	0.515			
Prosthetic valves or intracardiac devices	(No/yes)	2.43	1.22–4.82	0.011			
central venous catheters inserted	(No/yes)	2.69	0.85–8.55	0.094			
**Laboratory tests**
Blood culture	(Negative/positive)	1.02	0.57–1.83	0.937			
Leukocyte count(×10^9^/l)	(4–10 / Else)	1.13	1.06–1.21	<0.001	2.46	1.12–5.36	0.024
Anemia(g/l)	(≥90/ <90)	1.52	0.81–2.86	0.198			
Platelet count(×10^9^/l)	(100–300 / Else)	1.38	0.74–2.59	0.315			
CRP (mg/l)	(<8.2 mg/l /≥8.2 mg/l)	2.20	0.75–6.46	0.154			
PCT (ng/ml)	(<0.5 ng/ml/≥0.5 ng/ml)	1.73	0.97–3.10	0.063			
**Echocardiography**
Left-sided IE	(No/yes)	2.06	1.05–4.03	0.035	2.92	1.20–7.13	0.019
Right-sided IE	(No/yes)	2.56	0.96–6.83	0.060	6.58	1.63–26.53	0.008
**Duration of Antibiotic use**	(≥6w / <6w)	2.19	1.11–4.31	0.024			
**Surgery**	(Yes/no)	20.04	9.88–40.65	<0.001	18.94	8.18–43.86	<0.001

*IE, Infective endocarditis; CRP, C-reactive protein; PCT, Procalcitonin*.

### Nomogram Prediction Model for In-hospital Mortality in IE

**Figure 2:**
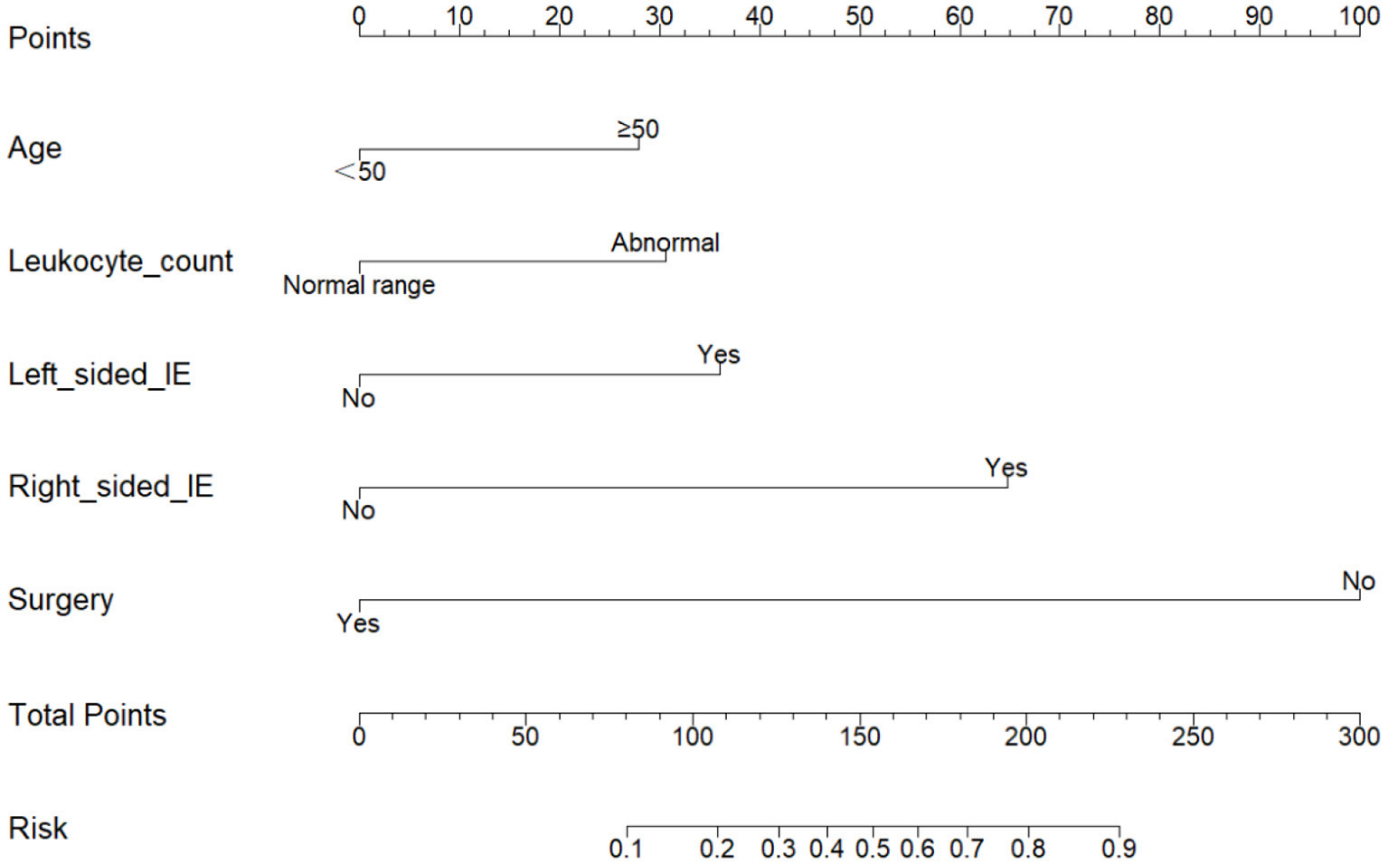
Nomogram for predicting in-hospital mortality in patients with infective endocarditis. To estimate in-hospital mortality in patients with IE, mark patient values at each axis, draw a straight line perpendicular to the point axis, and sum the points for all variables. Next, mark the sum on the total point axis and draw a straight line perpendicular to the risk axis.

**Figure 3:**
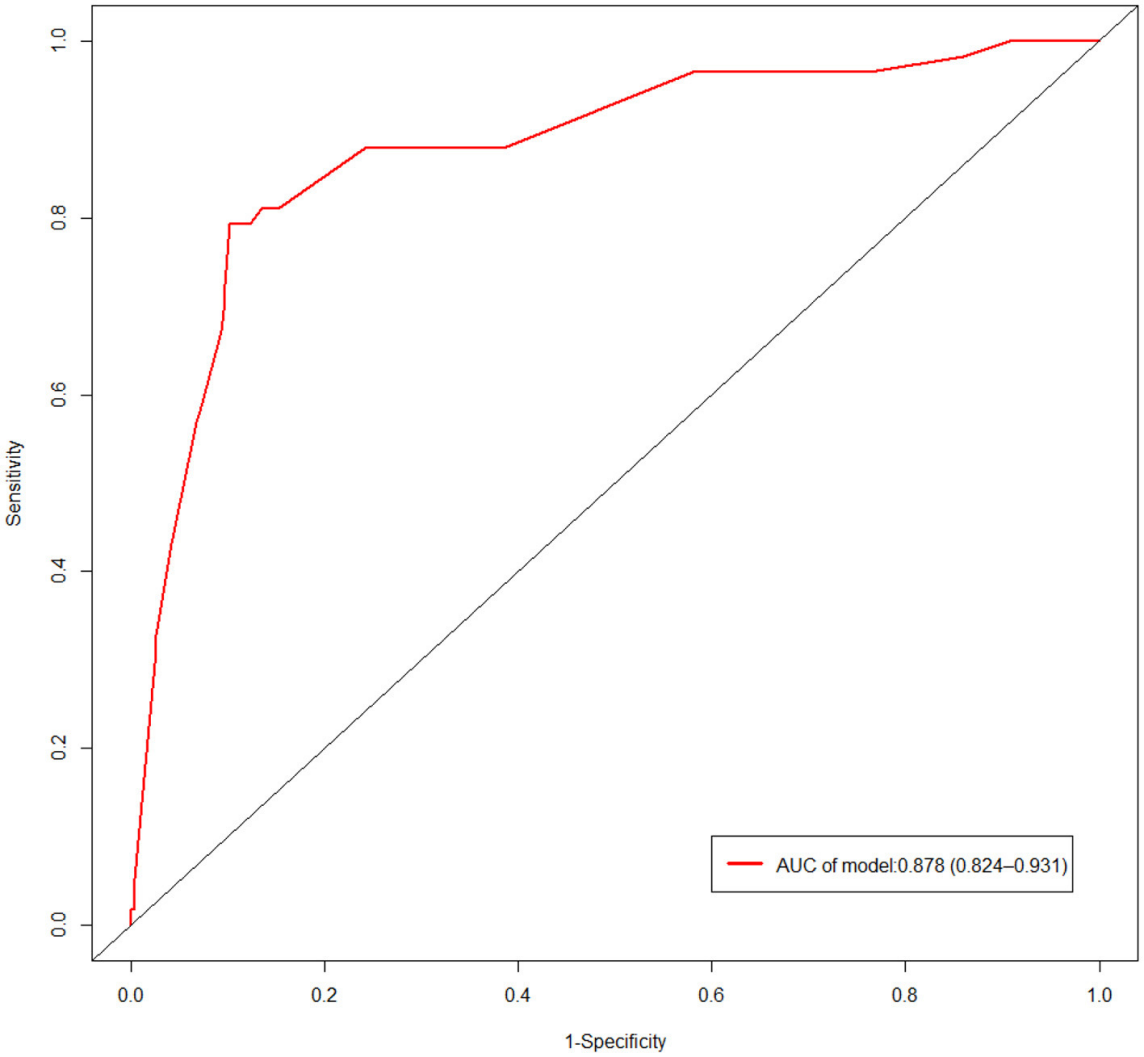
Receiver operating characteristic curve of the prediction model. AUC, Area Under Curve.

In the multivariate logistic regression analysis, factors that were statistically significant were used to create a risk prediction model for in-hospital mortality in IE, which was displayed as a nomogram (Figure 2). The area under the Receiver Operating Characteristic (ROC) curve is equivalent to the C-index in dichotomous logistic regression, therefore ROC curve analysis was applied to test the model's predictive performance, providing an Area Under Curve (AUC) of 0.878 (95%CI: 0.824–0.931) (Figure 3). The bootstrap validation method was used to perform internal validation of the prediction model, and the results revealed that the model prediction accuracy was 0.852 and the Kappa value was 0.53. The results of the Hosmer-Lemesho goodness-of-fit test of the model showed χ^2^ = 2.919, *P* = 0.232, indicating that the model fit was good.

**Figure 4:**
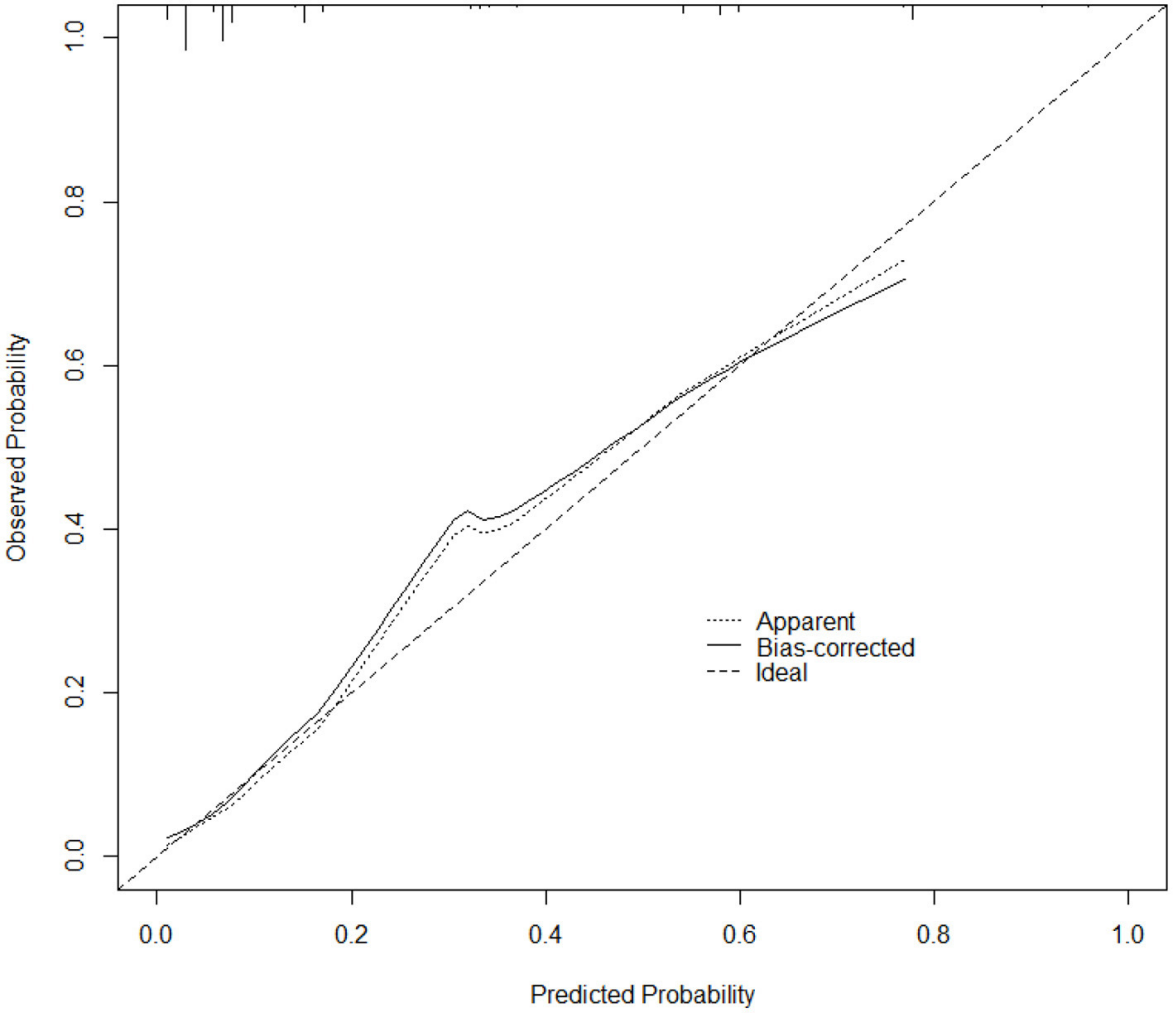
Calibration curve of the nomogram prediction model for IE. The *x*-axis depicts the predicted probability of dying during hospitalization, whereas the y-axis depicts the observed probability of dying during hospitalization.

To further verify the validity of the model, the calibration curve was established using R software (Figure 4), and the figure shows that the calibration curve of the model fits well with the standard ideal curve. Based on the simple nomogram, we also created a dynamic nomogram, which can quickly generate predicted values of IE in-hospital mortality after inputting the clinical characteristics of IE study subjects.

## Discussion

IE is an uncommon infectious disease in clinical practice, but with the development of society and medical advances, the number of patients suffering from valve disease, prosthetic valve implantation, cardiac devices, hemodialysis, and receiving immunosuppressive therapy is increasing, eventually leading to a yearly increase in the incidence of IE, which is now the fourth most common life-threatening infectious syndrome after sepsis, pneumonia, and intra-abdominal abscess (2, 11). The incidence of IE continues to increase and the mortality rate of IE has also remained at a high level (14–22% in hospital and 40% at 1 year after discharge) (12, 13). In addition to the clinical characteristics of the patient and the presence of underlying cardiac disease, studies have shown that the prognosis of IE is also influenced by the type of causative microorganism, the location of the cardiac vegetation, and the type of valve involved (native valve or prosthetic valve) (9, 14). It was found that among the pathogenic bacteria in IE, *Staphylococcus aureus, Streptococcus griseus* and Enterococcus are the most frequently seen in IE, accounting for about 70% of all (15). The mitral and aortic valves were reported to be the most commonly involved in IE patients with valve involvement (12). When compared to native valve endocarditis (NVE), prosthetic valve endocarditis (PVE) has a higher clinical death rate. PVE is also an independent risk factor for the prognosis of IE (14). The key to reducing the clinical mortality of IE patients is to immediately identify high-risk IE patients through comprehensive examination of early clinical characteristics, and then to use mature anti-infective medication therapy and suitable surgical intervention. To achieve this goal, physicians urgently need an adequate statistical prediction model to quantify the likelihood of clinical death in IE patients.

Anti-infective medication therapy is the foundation of the treatment of IE in clinical practice, and mature drug treatment programs can enhance the effectiveness of anti-infective therapy (16). According to the traditional approach, the strategy of using IE anti-infective medications is to start treatment early, with a large dose, and for a long period of time, with anti-infective treatments normally taking 4–6 weeks to work. Surgical treatment for IE patients began in the early 1960s and has since evolved into a standard treatment for IE (17). The proportion of patients treated surgically in our study was 74.1%. In today's IE treatment, the duration of anti-infective medicine and whether or not to treat surgically have become key variables influencing IE patients' in-hospital mortality (18). Therefore, in this study, the duration of anti-infective medication use during the IE hospitalization and the adoption of surgical treatment were also included together as two key variables in the construction of the statistical prediction model.

We analyzed the data from the cured and dead groups to see whether there were any risk factors that could have influenced the study's outcomes. After using univariate and multivariate logistic regression, we discovered that age (OR = 2.28, 95%CI 1.01–5.15, *P* = 0.048), abnormal leukocyte count (OR = 2.46, 95%CI 1.12–5.36, *P* = 0.024), left-sided IE (OR = 2.92, 95%CI 1.20–7.13, *P* = 0.019), right-sided IE (OR = 6.58, 95%CI 1.63–26.5, *P* = 0.008) and without surgery (OR = 18.94, 95% CI 8.18–43.86, *P* < 0.001) were independent risk factors for mortality during hospitalization in patients with IE. We created a statistical prediction model based on the five variables listed above and displayed it as a nomogram (Figure 2).

In order for the model to be used in the clinic, the accuracy of the model prediction is our primary concern. Therefore, after the nomogram prediction model was established, we evaluated the diagnostic performance of the model. The evaluated C-index of the model was found to be 0.878 (95% CI: 0.824–0.931). Internal validation of the prediction model using bootstrap validation method revealed that the prediction accuracy of the model was 0.852 and the kappa value was 0.53, which showed that the prediction accuracy of the model was high. In essence, the nomogram prediction model represents a scientific statistical method that can theoretically predict the probability of occurrence of any positive event in the clinic. Many oncologists currently use the nomogram prediction model to predict the prognosis of cancer patients and are also increasingly being used to predict the occurrence of clinical adverse events in non-oncology patients (4, 5, 10). One study established a Nomogram prediction model to predict the incidence of sternal incision problems in patients with median thoracotomy. The C-index of the model was 0.705, indicating that the Nomogram prediction model could accurately predict the occurrence of postoperative sternal incision problems (5). The findings of these studies suggest that the nomogram prediction model can accurately predict the incidence of positive clinical events in patients.

Among the five independent risk factors obtained from multifactorial logistic regression analysis, the age factor was a common clinical variable affecting survival outcome, and patients with IE ≥50 years of age will have a higher risk of in-hospital death compared to those <50 years of age (OR = 2.28). As an infectious condition, IE is frequently accompanied by a rise in white blood cell counts, and individuals with severe infections will develop leukopenia as a result of bone marrow suppression. Blood screening parameters could predict adverse outcomes and in-hospital mortality in IE, according to a study by Li et al. The neutrophil-to-platelet ratio (NPR) from routine blood tests at IE patients' admission allowed risk stratification and prediction of in-hospital mortality in IE patients, and the results showed that the prediction model using NPR as the main variable performed well in predicting in-hospital mortality in IE patients. Evaluation with the ROC revealed an AUC of 0.832 (19). In our study, patients with abnormal leukocyte count had a higher risk of death than those with normal leukocyte count (OR = 2.46).

TTE was performed on all of the individuals, while TEE was performed on some of the patients. TTE was our major method of locating cardiac vegetation in our study. When there is cardiac vegetation on the valve, the valve function will be seriously affected, resulting in valve insufficiency and movement disorders. Furthermore, pathogens wrapped in cardiac vegetation can produce toxins and enzymes, and these toxins and enzymes can cause tissue disintegration and invasion, which can lead to serious complications, such as heart failure caused by valve reflux or fistula, and the damaged tissue cannot regenerate, resulting in irreversible heart damage (20). Exfoliation of cardiac vegetation can cause embolism and other significant consequences in addition to direct injury to the attached myocardium. Embolism events produced by cardiac vegetation detachment have been revealed to be the main cause of poor prognosis and increased mortality in IE (21). Systemic embolism can result from the loss of left-sided IE cardiac vegetation, and all arteries may be affected. The most prevalent clinical embolism event in IE patients is embolic stroke, which is often associated with a poor prognosis (22). In our study, 193 (65.6%) patients had left cardiac vegetation (OR = 2.92) and 19 (6.5%) patients had right cardiac vegetation (OR = 6.58). Both left-sided IE and right-sided IE were associated with higher in-hospital mortality compared with patients without cardiac vegetation. During the treatment of IE, there are cases in which the infection is not controlled despite the identification of the pathogen by blood culture and the prolonged and continuous application of sensitive antimicrobial drugs. Recent research has provided a plausible explanation for this phenomenon, the concept of biofilms, it refers to bacterial populations living embedded in a self-produced extracellular polysaccharide slime-like matrix, and it will protect bacteria from host immune defenses and impedes antimicrobial efficacy. Biofilms protect bacteria from the immune system of the host and reduce the efficacy of antimicrobial medications, making it difficult to eradicate infection with drug therapy alone (23). In order to reverse this scenario, surgical treatment to remove infected lesions from heart tissue is required.

Approximately half of the patients with IE require surgical treatment due to severe complications (20). Currently, surgery is a common treatment for IE, and the indications for surgery are well agreed upon. Patients with signs of heart failure, severe valve dysfunction, PVE, paravalvular abscess or myocardial fistula invasion, recurrent systemic embolism, large mobile cardiac vegetation, and persistent sepsis after more than 5–7 days of adequate antibiotic therapy should be considered for surgical treatment (2). The majority of patients with IE are accompanied by the development of cardiac vegetation. In our study, 202 individuals (68.7%) were discovered to have cardiac vegetation during the early examination by TTE and TEE (193 left-sided vegetation, 19 right-sided vegetation, and 10 bilateral vegetation). In 262 (89.1%) of the 294 research participants, surgical exploration or recurring echocardiographic examination during hospitalization revealed cardiac vegetation. Surgery is beneficial for a variety of reasons. First, infected lesions in the heart or valve tissue can be entirely removed with direct-vision cardiac surgery. Second, while antimicrobial medications can prevent future structural damage to the heart, they cannot restore the integrity of injured tissue or valves, whereas surgery can restore valve function as well as the heart's structural integrity. Third, microbiological investigation of surgically excised tissue gives clinicians another chance to identify bacteria and assess their antibiotic susceptibility, and microbiological examination of surgical materials is especially useful in IE with negative blood cultures. Fourth, the most common preventable complication of IE is embolic stroke, and surgical cardiac vegetation removal can effectively prevent embolic occurrences (2, 24, 25). The left-sided IE and the right-sided IE serve as key variables in the nomogram prediction model we developed, and we can simulate different situations that may occur in IE patients in the clinic by assigning them different values, and the impact of these different conditions on the in-hospital mortality of IE is presented directly by the probabilities predicted by the model.

In terms of the timing of surgery, the traditional view is that patients with IE need 4–6 weeks of regular anti-infection treatment with normal temperature and negative blood cultures before surgery to reduce the probability of postoperative recurrence. However, during this time, patients may experience severe structural damage such as valve regurgitation and perforation, and may also develop heart failure and other manifestations as a result. Furthermore, some patients may develop embolic events as a result of cardiac vegetation dislodgement during therapy, as well as an increase in-hospital mortality. As a result, a growing number of experienced groups are actively advocating for early surgery rather than waiting for complications to appear (2, 3, 26). Although there is no clear definition of early surgery in terms of the timing of surgery, a growing number of studies have confirmed that early surgery reduces in-hospital mortality, follow-up mortality, and IE-related mortality in patients and does not increase the probability of recurrent infection (27–29).

**Figure 5:**
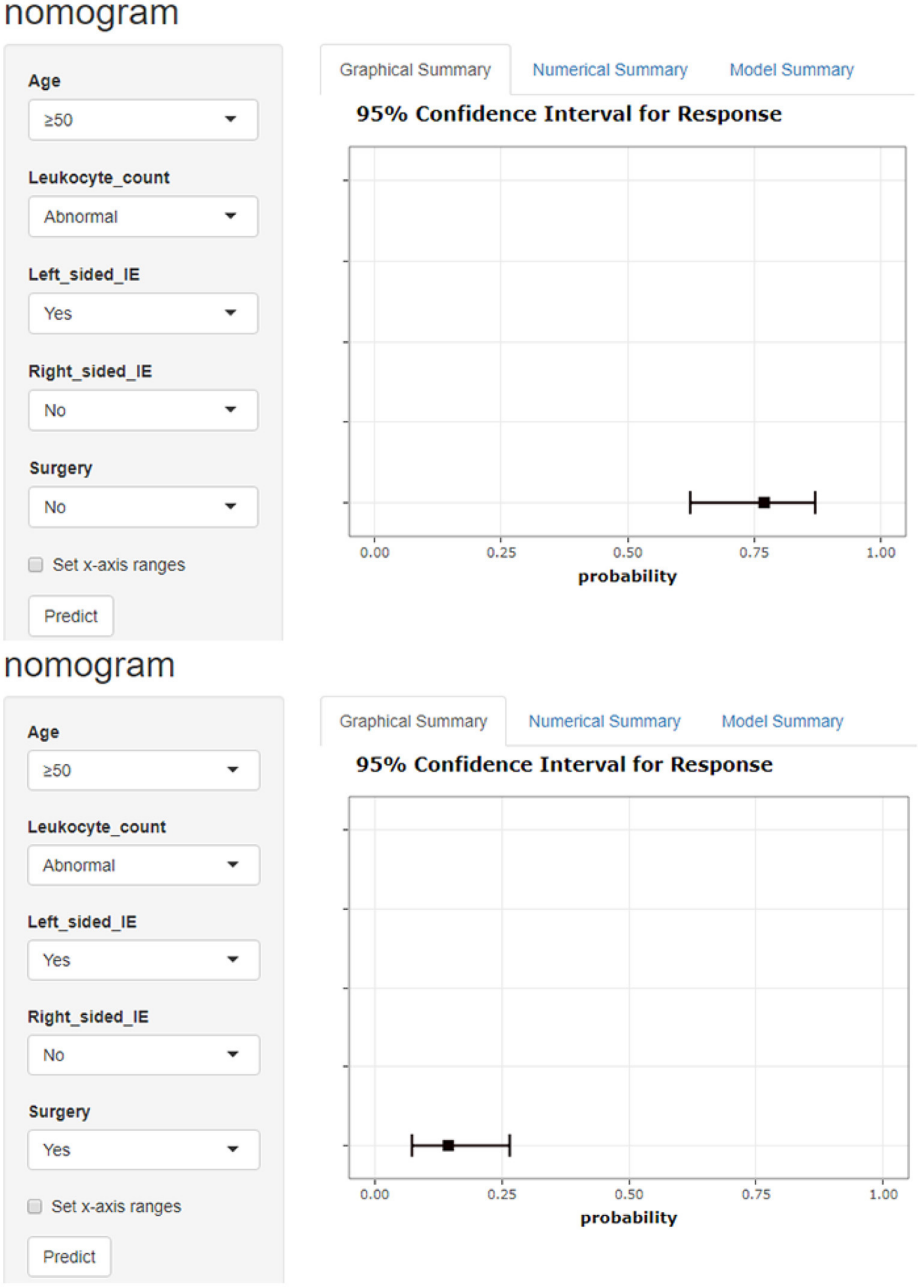
Model-based dynamic nomogram. Entering a patient's early clinical characteristics into a dynamic nomogram can quickly calculate the probability of in-hospital death. The picture shows a patient over 50 years old with abnormal leukocyte count and left-sided IE. The in-hospital mortality rate was 77.0% without surgery and 14.5% with surgery.

In our study, 218 (74.1%) of the 294 study participants had surgical treatment. Our cardiac surgeons all used a median sternotomy surgical technique under general anesthesia, created extracorporeal circulation on a regular basis, and chose different surgical procedures depending on the patient's situation. On the basis of entirely removing the infected tissue in the heart, the principle of surgery is to reconstruct the heart structure to the greatest extent possible and restore normal heart function. One hundred and sixty five patients (75.7%) had valve replacement surgery, 36 (16.5%) had valve repair surgery, and 17 (7.8%) had congenital heart disease correction surgery. In our statistical prediction model, the absence of surgical therapy (OR = 18.94) was an independent risk factor for in-hospital mortality in IE. Patients receiving surgery had a 24.1% in-hospital mortality rate, while patients without surgery had a 75.9% in-hospital mortality rate. We can make a dynamic nomogram (Figure 5) based on a statistical prediction model, which can calculate the in-hospital mortality rate of IE by inputting the patient's early clinical features and surgical option into the model. The probability predicted by the model can intuitively tell doctors and patients the harm caused by IE and the benefits of surgical treatment.

In conclusion, the nomogram prediction model we developed performed well, with a C-index of 0.878 (95%CI: 0.824–0.931) and the internal validation of the model by bootstrap validation method showed a prediction accuracy of 0.852 and a bootstrapped-concordance index of 0.53. The development of dynamic nomogram could facilitate the use of statistical prediction models in clinical practice. The algorithm can accurately forecast IE patients' in-hospital death rate, and doctors can use the anticipated likelihood to make treatment decisions.

### Limitations

First, some patients were transferred to our hospital after having care in another hospital, resulting in the loss of some clinical information. In addition, this is a retrospective study, and retrospective analysis inevitably results in bias in the study design and case selection. Finally, because this is a single-center study, the epidemiology and clinical characteristics of all IE in the region cannot be accurately represented. When applied to IE patients at other centers, the statistical prediction model built on clinical data of IE patients obtained in this center may have a lower prediction accuracy. To develop the statistical prediction model, we will need to collect clinical data from IE patients from several centers.

## Conclusion

Comprehensive assessment of early clinical characteristics of patients can help doctors identify high-risk IE patients early. In this study, we found that age, abnormal leukocyte count, cardiac vegetations and the absence of surgical treatment were independent predictors of in-hospital mortality in IE patients. This Nomogram prediction model, which is based on IE patients' early clinical features and surgical options, has good diagnostic performance and clinical application potential.

## Data Availability Statement

The raw data supporting the conclusions of this article will be made available by the authors, without undue reservation.

## Ethics Statement

The studies involving human participants were reviewed and approved by Ethics Committee of Fujian Provincial Hospital. Written informed consent to participate in this study was provided by the participants' legal guardian/next of kin.

## Author Contributions

Z-JY and ZD drafted the manuscript. JL and Z-JN participated in the data collection. All authors were involved in the design of the study and approved the final version of the manuscript.

## Conflict of Interest

The authors declare that the research was conducted in the absence of any commercial or financial relationships that could be construed as a potential conflict of interest.

## Publisher's Note

All claims expressed in this article are solely those of the authors and do not necessarily represent those of their affiliated organizations, or those of the publisher, the editors and the reviewers. Any product that may be evaluated in this article, or claim that may be made by its manufacturer, is not guaranteed or endorsed by the publisher.
